# Primary scrotal Paget’s disease: a case report and literature review

**DOI:** 10.3389/fmed.2025.1680986

**Published:** 2026-01-16

**Authors:** Pinyao Liang, Junfeng Zhang, Xiaofeng Xie, Yumin Wang, Junxiong Li, Jingbo Qin, Peng Gu, Xiaodong Liu

**Affiliations:** 1 Second Department of Urology, The First Affiliated Hospital of Kunming Medical University, Kunming, Yunnan, China; 2 Department of Dermatology, The First Affiliated Hospital of Kunming Medical University, Kunming, Yunnan, China

**Keywords:** case report, extramammary Paget’s disease, primary, radiotherapy, scrotal Paget’s disease, wide local excision

## Abstract

**Objective:**

The objective of the study was to report a case of primary scrotal Paget’s disease and discuss its clinical characteristics, diagnosis, and management based on the literature.

**Methods:**

The clinical data of a patient with primary scrotal Paget’s disease admitted to the Second Department of Urology at the First Affiliated Hospital of Kunming Medical University in December 2024 were retrospectively analyzed.

**Results:**

This is a case report of a 58-year-old man who presented with erythema on the left penile root and scrotum persisting for over 6 months. Histopathological examination of the skin lesion confirmed extramammary Paget’s disease. He underwent wide local excision under general anesthesia. The surgery was successful, resulting in negative surgical margins. Postoperative adjuvant radiotherapy was administered due to the diffuse nature of the lesion boundaries. The patient tolerated the treatment well, recovered favorably, and is currently on a scheduled follow-up regimen with no evidence of recurrence to date.

**Conclusion:**

The early clinical manifestations of scrotal Paget’s disease resemble chronic dermatoses. Early diagnosis and exclusion of metastatic disease or underlying internal malignancies are crucial. The primary treatment is wide local excision, with radiotherapy serving as adjuvant therapy post-surgery.

## 1 Introduction

Extramammary Paget’s disease (EMPD) is a rare cutaneous malignancy, typically occurring in apocrine gland-rich areas such as the vulva, penis, scrotum, anus, perianal region, and axilla. Scrotal Paget’s disease, a subtype of EMPD, predominantly affects middle-aged and elderly individuals. Due to its early clinical similarity to conditions such as eczema, dermatitis, and fungal infections, it is often misdiagnosed. We report a case of scrotal Paget’s disease admitted to the Second Department of Urology at the First Affiliated Hospital of Kunming Medical University in December 2024. The patient underwent successful wide local excision under general anesthesia, followed by adjuvant radiotherapy. Currently, the patient is undergoing scheduled follow-ups every 3 months, with no signs of recurrence observed to date. This case is reported as follows.

## 2 Clinical data

### 2.1 General information

**Figure 1:**
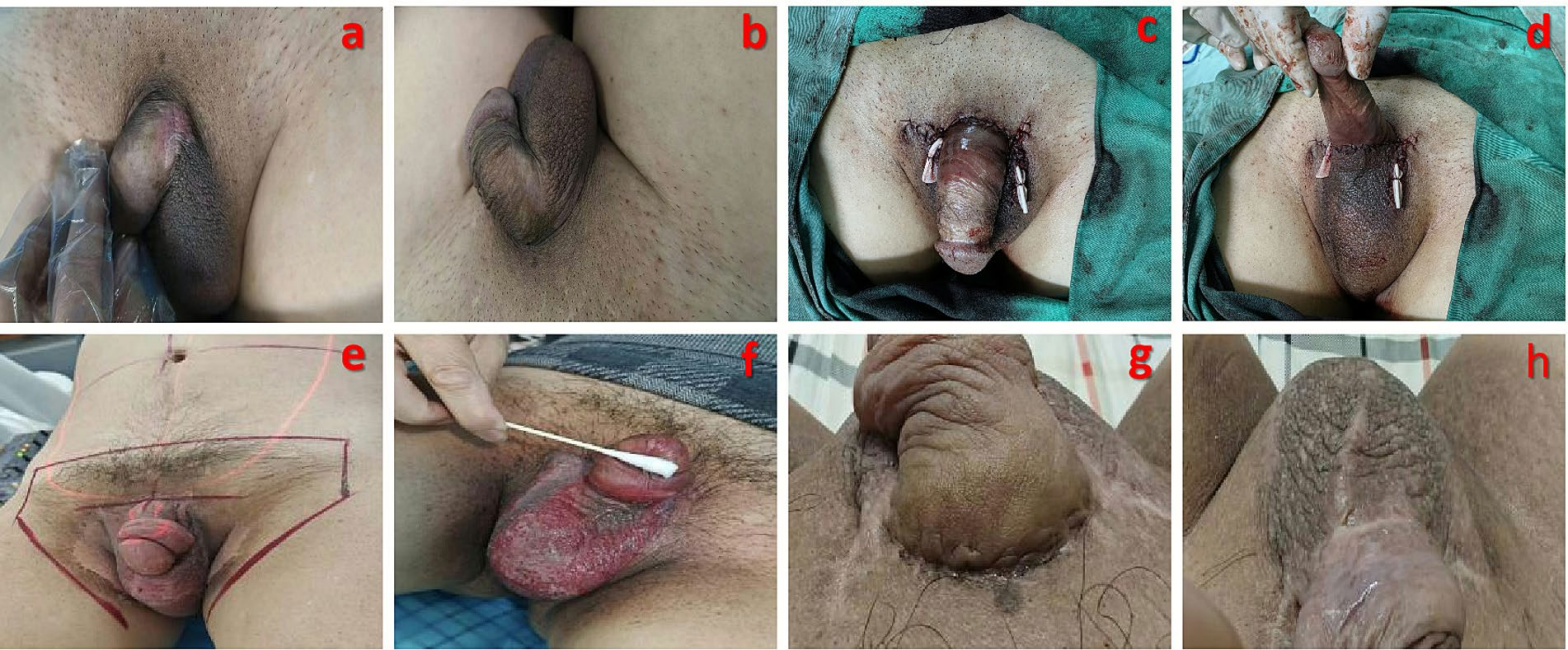
Overview of patient’s clinical course. **(a)** Erythema on the left penile root and scrotum at initial presentation (3 × 2 cm). **(b)** Disease progression with similar erythema appearing on the right penile root and scrotum (2 × 1 cm). **(c,d)** Postoperative appearance after wide local excision of the scrotum. **(e)** Radiation field for postoperative radiotherapy. **(f)** Grade III radiation dermatitis observed during radiotherapy. **(g,h)** Good healing of the surgical wound and resolved radiation dermatitis at the end of treatment.

A 58-year-old man was admitted on 16 December 2024 due to “erythema on the left penile root and scrotum persisting for over 6 months.” Approximately 6 months prior, the patient developed erythema on the left penile root and scrotum (Figure 1a) without obvious cause. The lesion exhibited scaling but no erosion, ulceration, or exudate. The patient reported no significant symptoms such as pruritus or pain. Conservative treatment at another hospital was ineffective, and the skin lesion progressed, with similar erythema appearing on the contralateral penile root and scrotum (Figure 1b), leading the patient to seek consultation at the Dermatology Department of our hospital.

**Figure 2:**
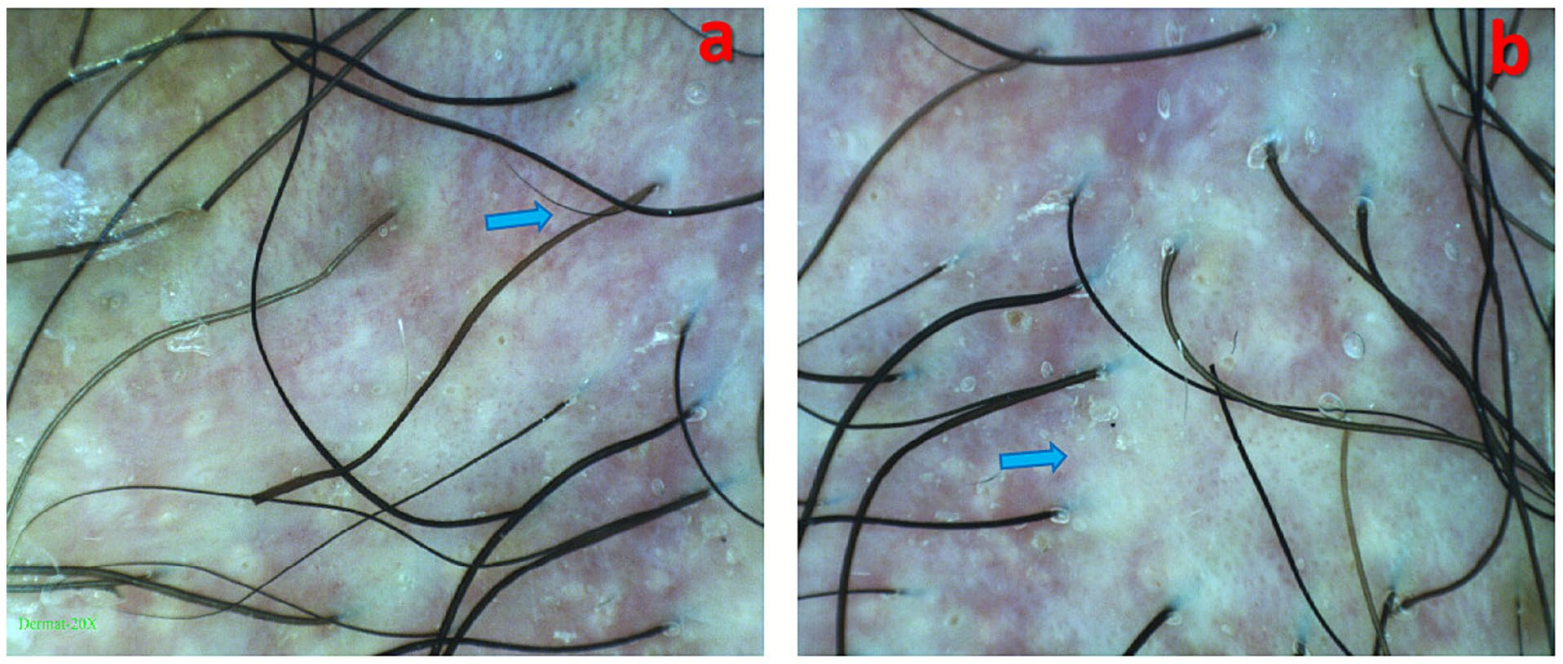
Dermoscopic view on a pink background reveals atypical vascular structures, along with: **(a)** radial white linear structures (arrow); **(b)** white structureless areas (arrow).

**Figure 3:**
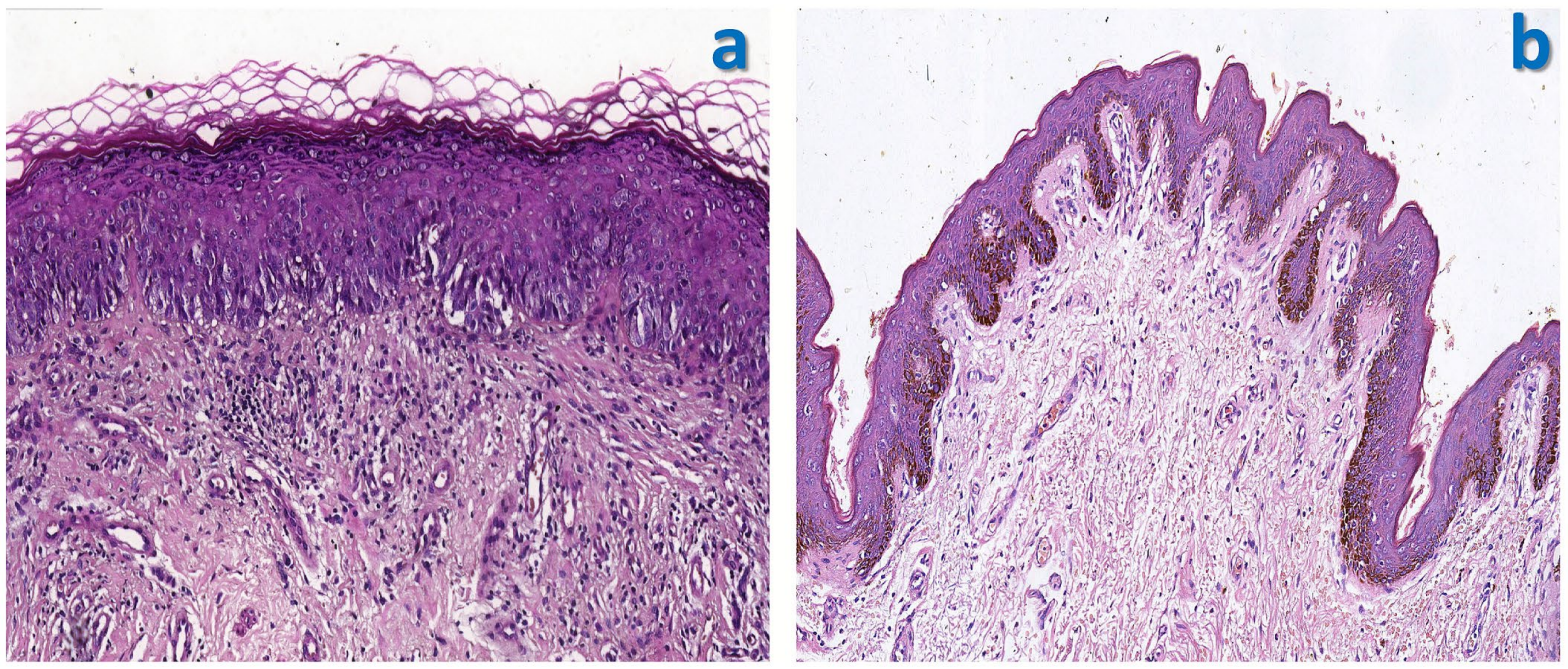
Histopathological examination of the scrotal lesion (H&E staining). **(a)** Preoperative biopsy specimen (×400): Numerous Paget cells are observed within the epidermis, showing large cell size, abundant pale cytoplasm, and prominent nucleoli; the tumor cells are confined to the epidermis, with no dermal invasion observed. **(b)** Widely excised postoperative specimen (×200): The epidermis and base of the marginal skin show no tumor cell involvement.

**Figure 4:**
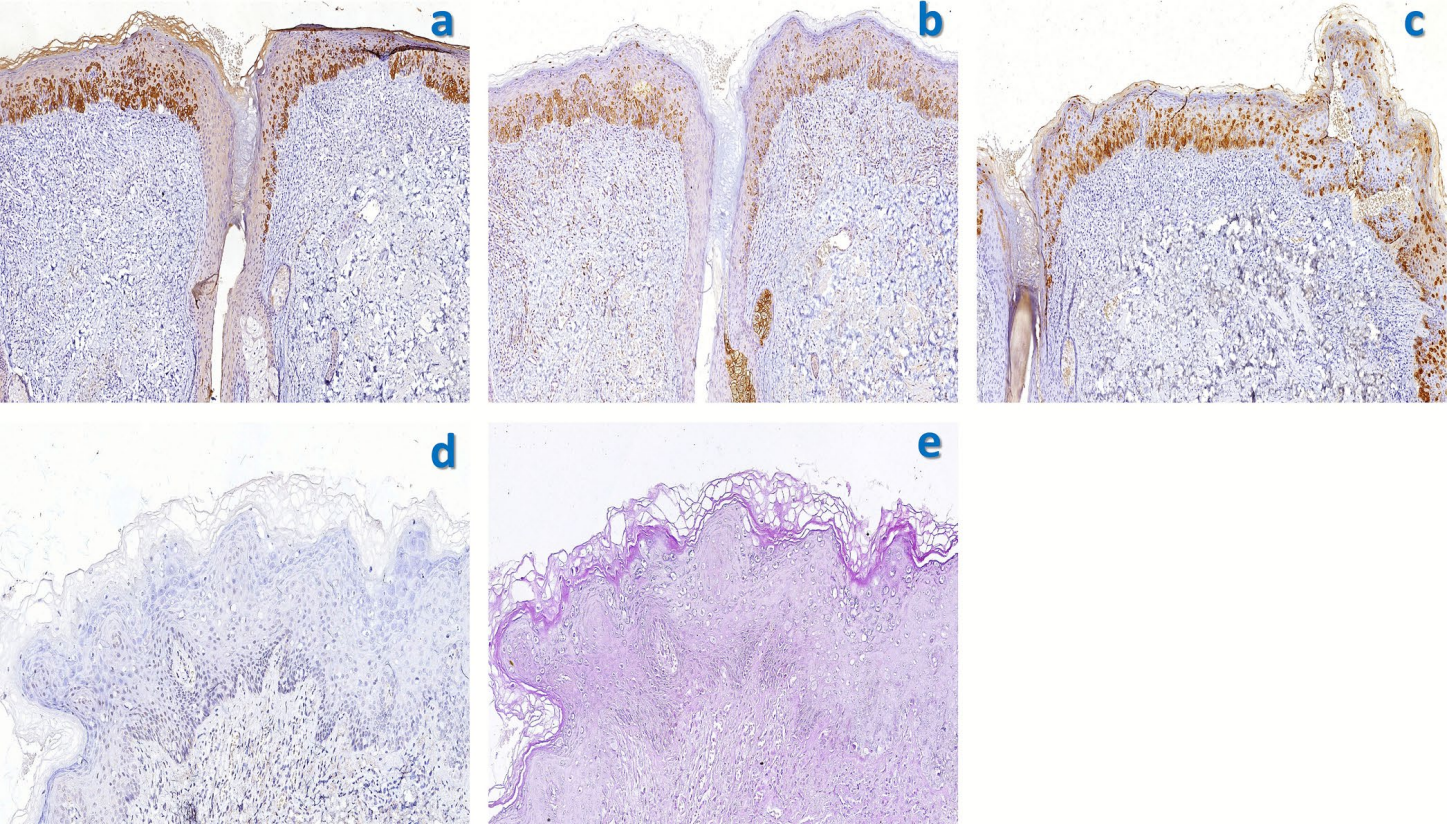
Immunohistochemical staining of the scrotal lesion. **(a)** CK7(+) cytoplasmic staining (×100). **(b)** EMA(+) membranous pattern (×100). **(c)** CEA(+) diffuse reactivity (×100). **(d)** CDX-2 is negative (×200). **(e)** PAS staining (×200) shows no definite mucin-positive material within the tumor cell cytoplasm. IHC was performed with diaminobenzidine chromogen and hematoxylin counterstain.

Upon admission, a dermatological evaluation was performed. Dermoscopy revealed a pinkish background with focally distributed dotted, globular, and glomerular vessels, accompanied by white reticulated streaks (Figure 2). Fungal culture was negative. To confirm the diagnosis, a pathological biopsy of the skin lesion was conducted. The results showed parakeratosis, mild acanthosis, liquefaction degeneration of the basal layer, and numerous Paget cells infiltrating the dermis (Figure 3a). Immunohistochemical staining was positive for CK7, EMA, and CEA but negative for CK20 and P63 (Figure 4). Based on these findings, a pathological diagnosis of “extramammary Paget’s disease” was established, and the patient was subsequently transferred to the Second Department of Urology for further management.

The patient had a past medical history of hemorrhoids. In 2019, a colonoscopy at our hospital identified a polyp (0.6 cm in diameter) in the transverse colon, with pathology suggesting tubular adenocarcinoma, which was subsequently removed through endoscopic resection. One year later, a follow-up colonoscopy revealed a broad-based sessile polyp (0.7 cm in diameter) near the hepatic flexure of the transverse colon. Pathological examination of the resected polyp again indicated early tubular adenocarcinoma. No further intervention was undertaken thereafter. A subsequent follow-up colonoscopy in 2023 showed only the presence of circumferential internal hemorrhoids.

### 2.2 Auxiliary examinations and diagnosis

Routine blood and biochemical tests showed no significant abnormalities. Tumor markers, including carcinoembryonic antigen (CEA), cytokeratin 19 fragment (CYFRA-21-1), and prostate-specific antigen (PSA), were within normal limits. Ultrasound of the urinary system and superficial lymph nodes was unremarkable. Fluorine-18 fluorodeoxyglucose positron emission tomography–computed tomography (F-18 FDG PET-CT) revealed slightly thickened skin at the penile root and bilateral scrotum with diffusely mildly increased glucose metabolism, consistent with a malignant lesion, and slightly thickened skin at the left perianal margin with mildly increased glucose metabolism, suggesting possible tumor involvement. No other areas of abnormal FDG uptake were detected elsewhere in the body. Repeat colonoscopy showed internal hemorrhoids; the terminal ileum and colorectal mucosa were normal.

### 2.3 Treatment and outcome

The patient underwent wide local excision of the scrotum under general anesthesia (Figures 1c,d). Following anesthesia induction, the patient was placed in the lithotomy position. The area was sterilized and draped, and a urinary catheter was inserted. An electrocautery device was used to excise the lesion with a 1-cm margin around the visible edge, extending down to the deep fascia. Intraoperative frozen section analysis indicated positive surgical margins. Consequently, the excision was extended outward beyond the initial margins until frozen sections confirmed negative margins. Postoperative hematoxylin and eosin (H&E) staining showed no Paget cell involvement of the epidermis or base; margins and base were negative (Figure 3b). The findings of immunohistochemistry revealed CK7(+), CK20(−), CDX-2(−), and CK18(+). These findings were consistent with the preoperative biopsy. As the postoperative pathology clearly demonstrated that the Paget cells were confined to the epidermis without invasion of the basement membrane, a definitive diagnosis of *in situ* extramammary Paget’s disease was established.

Following evaluation by a multidisciplinary team, postoperative adjuvant radiotherapy was decided upon to reduce the risk of recurrence. The patient completed radiotherapy at Yunnan Cancer Hospital from 15 February to 28 April 2025 (Figure 1e), with a total cumulative dose of 70 Gy in 35 fractions. The specific regimen was as follows: Phase 1 (starting 15 February): 50 Gy F; Phase 2 (starting 25 February): an additional 10 Gy/5 F to the tumor bed area; and Phase 3 (starting 22 April): a boost of 10 Gy/5 F using 8 MeV electron beams. During radiotherapy, the patient developed Grade III radiation dermatitis (Figure 1f), which improved after temporarily suspending radiotherapy and providing symptomatic supportive treatment, including anti-infection measures, skin care, and analgesics. The patient subsequently completed the entire treatment course.

One month after the final radiotherapy session, follow-up examinations through ultrasound, CT, and MRI showed no evidence of tumor recurrence. The surgical site and the radiation dermatitis had healed well (Figures 1g,h). The patient expressed satisfaction with the treatment process and outcome. Currently, the patient is undergoing scheduled follow-ups every 3 months, with no signs of recurrence observed to date.

## 3 Discussion

EMPD is a rare cutaneous malignancy, accounting for approximately 6.5% of all Paget’s disease cases (1). It most commonly affects individuals aged 50–80 years (2, 3), with a higher prevalence among Asian males and Caucasian females in Western countries (4). Herrel et al. (5) reported the highest incidence of EMPD in Asian males, four times greater than in Caucasian males, while the incidence in Black males was lower than in both Asian and Caucasian males. Scrotal Paget’s disease, as a form of EMPD, has an estimated incidence in China of approximately 0.4 per 1,000,000 (6), compared to approximately 0.7 per 1,000,000 in Europe (7).

EMPD, also known as eczematoid carcinoma, most frequently involves the vulva in females and the scrotum in males (3, 8, 9). The primary clinical presentation includes scaly, erythematous, eczematous lesions at the affected site (10). Pruritus is the most common symptom, affecting approximately 70% of patients; others may experience pain, bleeding, or swelling. Approximately 10% of patients are asymptomatic (11). The average time from symptom onset to diagnosis is approximately 2 years (3). In addition, the early resemblance of EMPD to chronic dermatoses, coupled with the often-private location of lesions, frequently leads to delayed patient presentation or insufficient physician examination, increasing diagnostic difficulty. In a retrospective study of EMPD patients, the disease duration ranged from 1 month to 30 years (median: 3 years). Initially, 11 patients (14.7%) were misdiagnosed, with 7 of them being misdiagnosed with eczema (12). Therefore, EMPD should be considered, and a skin biopsy promptly performed, when erythematous lesions resembling chronic inflammatory or infectious skin conditions fail to respond to conventional treatments (especially potent topical steroids or antifungals) or recur persistently.

Wilkinson et al. (13) classified vulvar Paget’s disease as primary or secondary based on origin, each with three subtypes. Primary EMPD is typically categorized as intraepidermal cutaneous Paget’s disease *in situ*, invasive intraepidermal cutaneous Paget’s disease, and intraepidermal cutaneous Paget’s disease. Secondary EMPD includes anorectal-origin Paget’s disease, urothelial-origin Paget’s disease, and Paget’s disease from other origins. Furthermore, Chanda et al. (14) found a significant association between EMPD and coexisting internal malignancies, suggesting that EMPD may act as a cutaneous marker for such malignancies. Consequently, it is recommended that EMPD patients undergo comprehensive evaluation, particularly including F-18 FDG PET-CT, to fully assess the disease extent, exclude metastatic lesions, and detect any underlying malignancies (15). In clinical diagnosis, EMPD must be differentiated from various benign and malignant conditions. At the clinical level, its lesions should be distinguished from chronic eczema, contact dermatitis, tinea cruris, and Bowen’s disease. The key clinical distinguishing feature is that typical EMPD lesions often present as unilateral, well-demarcated infiltrative erythema or plaques that respond poorly or not at all to conventional topical anti-inflammatory or antifungal therapies. Histopathology is the cornerstone of definitive diagnosis. The characteristic histopathological finding is the presence of scattered or nested Paget cells within the epidermis. These cells are large, with abundant pale-staining cytoplasm, and exhibit large nuclei with prominent nucleoli (16, 17). Immunohistochemical staining is the final and definitive step in the differential diagnosis. The typical immunophenotype of primary EMPD is characterized by CK7(+), CEA(+), GCDFP-15(+), and CK20(−), and periodic acid–Schiff (PAS) staining may be positive or negative (18). It is noteworthy that primary perianal EMPD typically demonstrates a negative expression for both CK20 and CDX-2. The most critical differential diagnosis lies in distinguishing between primary and secondary EMPD. The latter typically exhibits an immunophenotype of CK20 positivity and GCDFP-15 negativity. Its specific marker expression depends on the primary tumor origin; for instance, gastrointestinal origin often shows CDX-2 positivity, whereas urothelial origin may express uroplakin II/III (2). Utilizing this immunohistochemical profile allows for the systematic exclusion of other similar diseases: 1. melanoma: melanoma markers such as S-100, HMB-45, and MART-1 are positive, whereas they are negative in EMPD. 2. Squamous cell carcinoma and Bowen’s disease: both are typically positive for P63, a pattern opposite to that of EMPD. 3. Pagetoid spread of cutaneous adnexal carcinoma: this category encompasses malignancies originating from skin appendages. Among these, eccrine adenocarcinoma with epidermotropism is the most representative type. This lesion can closely mimic EMPD both morphologically and immunophenotypically (CK7 + and CEA+). Such a tumor often arises from the base of a poroma, presenting as an asymmetric carcinoma that broadly infiltrates the deeper dermis and subcutis. It is characterized by tumor formations with reticularly branched, ribbon-like structures that make broad-based contact with the surface epithelium. Similar to poroma, ductal differentiation and clear cell areas are possible. The tumor is composed of small basaloid cells with chromatin-rich nuclei and exhibits abundant mitoses (19). In contrast, Paget cells lack tonofibrils, are separated from surrounding keratinocytes by a clear halo, and do not undergo keratinization (20). Furthermore, cutaneous adnexal carcinomas are typically GCDFP-15-negative, which aids in distinguishing them from the majority of primary EMPD cases that are GCDFP-15-positive (21, 22). In this case, F-18 FDG PET-CT showed slight thickening and mildly increased glucose metabolism in the left perianal skin, raising suspicion of tumor involvement. However, perianal inspection and digital rectal examination were normal, and colonoscopy only revealed internal hemorrhoids. Tsai et al. (23), in a study of 156 hemorrhoid patients, found that eight patients (5.1%) had maximum FDG uptake higher than the maximum in normal controls. The authors proposed that the pathogenesis of hemorrhoids (inflammation, thrombosis, and vascular proliferation) might lead to abnormal FDG uptake on PET-CT. Given that our patient’s colonoscopy 5 years post-polypectomy showed only internal hemorrhoids, we attributed the increased glucose metabolism in that area more likely to benign vascular pathology associated with hemorrhoids rather than perianal Paget’s disease. To definitively confirm the diagnosis, supplementary immunohistochemical staining was performed. All additional stains were conducted on the paraffin-embedded tissue block from the preoperative diagnostic skin biopsy, which contained unequivocal tumor cells, ensuring the reliability of staining and assessment. The results revealed that CDX-2 was negative, providing key evidence against metastatic adenocarcinoma given the patient’s history, while PAS staining revealed no definite mucin-positive material within the tumor cell cytoplasm. Although the negative PAS staining necessitates consideration of pagetoid spread from a cutaneous adnexal carcinoma, this possibility is effectively ruled out by the histopathological and immunohistochemical findings. Histologically, the tumor cells are strictly confined to the epidermis, with no evidence of dermal invasion or involvement of adnexal structures confirmed on serial sections. Critically, the immunophenotype is definitive: the tumor cells are CK7(+), CK20(−), and CDX-2(−) and show strong positivity for GCDFP-15. This profile is highly indicative of primary EMPD and argues against both primary adnexal carcinoma and pagetoid spread from an underlying visceral adenocarcinoma. Combined with the H&E staining and immunohistochemistry results, which confirmed that the Paget cells were confined to the epidermis with no dermal invasion, the final postoperative diagnosis was established as *in situ* primary scrotal Paget’s disease.

The primary treatment for EMPD is radical surgery. To ensure the thoroughness of the procedure, precise preoperative assessment is crucial. Mapping biopsies serve as an important preoperative evaluation tool, accurately defining the true extent of the lesion and providing an objective basis for surgical resection. This is particularly significant for reducing the risk of postoperative recurrence, especially in cases with ill-defined borders or multiple lesions (24). Wide local excision is the standard surgical procedure, typically requiring a resection margin of 1–5 cm from the lesion edge (16, 25, 26). The extent of resection should be adjusted according to intraoperative frozen section pathology results until negative margins are achieved. Mohs micrographic surgery is another important surgical technique, particularly suitable for areas where tissue preservation is paramount. The formulation of treatment decisions and risk stratification heavily relies on prognostic assessment centered on tumor thickness. Studies have shown that tumor invasion depth (i.e., tumor thickness) and lymph node status are key prognostic indicators, closely associated with the risk of recurrence and metastasis (27–29). Consequently, in clinical evaluation, precise measurement of tumor thickness (vertical invasion depth) should be prioritized over merely assessing the clinically apparent lesion size (horizontal spread area). Clinical decisions should be guided more by the depth of invasion and clarity of tumor borders to achieve more individualized and precise treatment management (29). Based on the above principles, for patients with high-risk factors such as invasive disease, large tumor volume, or palpable lymph nodes, sentinel lymph node biopsy (SLNB) is an important assessment tool that provides crucial information for staging and prognosis (30). However, in this case, SLNB was not performed as preoperative imaging showed no abnormalities, and postoperative pathology confirmed the lesion was of the *in situ* type. For patients with postoperative high-risk factors (such as close to positive margins) or those who are unable to tolerate surgery due to advanced age or comorbidities, non-surgical treatment options need to be considered. These mainly include topical medications such as imiquimod (31) and 5-fluorouracil (32), radiotherapy, photodynamic therapy, and laser therapy. Among these, radiotherapy is the primary non-surgical treatment for invasive EMPD, especially suitable for patients with positive margins or those presenting with other high-risk features (30). A study by Guo et al. (12) of 75 EMPD patients found that none of the 15 patients who received postoperative radiotherapy experienced recurrence. The report by Hata et al. (27) further confirmed its efficacy: among 41 EMPD patients receiving radiotherapy, the local progression-free survival rates and disease-free survival rates were 88 and 55% at 3 years and 82 and 46% at 5 years, respectively. In this case, the final diagnosis based on pathology and immunohistochemistry was *in situ* primary scrotal Paget’s disease, with negative surgical margins. Given the diffuse nature of the lesion, postoperative adjuvant radiotherapy was ultimately administered to minimize the risk of local recurrence. The patient is currently undergoing regular follow-ups as planned, with no signs of recurrence to date; however, the long-term efficacy still requires continued observation.

## 4 Conclusion

Scrotal Paget’s disease is rare. Prompt diagnosis and treatment, coupled with comprehensive systemic evaluation upon diagnosis to exclude metastases and underlying malignancies, are essential. This approach significantly improves prognosis, potentially allows for more conservative surgical resection, minimizing impact on organ appearance and function, and may reduce the need for subsequent adjuvant therapies and overall healthcare burden. Furthermore, establishing a multidisciplinary team (MDT) approach and implementing a strict long-term follow-up protocol are instrumental in monitoring for recurrence and enhancing the quality of life.

## Data Availability

The original contributions presented in the study are included in the article/supplementary material, further inquiries can be directed to the corresponding author.
